# Long noncoding RNA CCAT2 as a novel biomaker of metastasis and prognosis in human cancer: a meta-analysis

**DOI:** 10.18632/oncotarget.18161

**Published:** 2017-05-24

**Authors:** Dailian Wang, Zhicong Chen, Haidan Xu, Anbang He, Yuchen Liu, Weiren Huang

**Affiliations:** ^1^ Key Laboratory of Medical Reprogramming Technology, Shenzhen Second People’s Hospital, The First Affiliated Hospital of Shenzhen University, Shenzhen, Guangdong, China; ^2^ Guangzhou Medical University, Guangzhou, Guangdong ,China; ^3^ Shantou University Medical College, Shantou, Guangdong, China; ^4^ Anhui Medical University, Hefei, Anhui, China

**Keywords:** CCAT2, lncRNA, cancers, lymph node metastasis, distant metastasis

## Abstract

Colon cancer-associated transcript2 (CCAT2), a long noncoding RNA (LncRNA), has been found to function as an oncogene in various cancers. However, the clinical value of CCAT2 in cancers remains unclear. Therefore, we performed this meta-analysis to investigate the association between CCAT2 level and metastasis & prognosis in malignant tumors. The meta analysis was performed by using a systematic search in PubMed, Web of Science, and Cochrane Library from inception to NOV 17, 2016. According to the inclusion and exclusion criteria,9 studies with 1084 patients were included in the meta-analysis.The result showed that overexpression of CCAT2 is positively correlated with lymph node metastasis (Odds ratio,OR=3.57, 95 % confidence interval(CI): 1.79-7.13, *p*<0.001) in a random-effects model (I^2^=71%, *p*=0.008) and distant metastasis(OR=7.68, 95 % CI: 3. 29-17.96, *p*<0.001) in a random-effects model (I^2^=41.9%, *p*=0.16).Likewise,we also found that high CCAT2 expression could predict unfavourable overall survival with pooled hazard ratio (HR) of 2.23 (95 % CI 1.68-2.96, *p*<0.00001) by a random-effects model (I^2^=37.5%, *p*=0.143) and poor metastasis-free survival in cancer patients (HR= 2.08, 95%CI:1.37-3.18 *p*=0.001) by a fixed-effects model (I^2^=0.0%, *p*=0.807). In conclusion,CCAT2 might be served as a novel molecular marker for predicting metastasis and prognosis in various human-cancers.

## INTRODUCTION

Cancer incidence and moratlity are increasing each year, making cancer a major public health problem and the leading cause of death worldwide. It is reported that4292,000 new cancer cases and 2814,000 cancer deaths are predicted to occur in China in 2015 [1].Currently, tumor patients can acquire benefit from comprehensive treatment strategies, such as surgery, chemotherapy, radiotherapy, targeted therapy and so on [2].Howerever, the clinical outcome of malignant tumor remains disappointed. One of the reasons affecting the tumor prognosis was lacking of effective early diagnostic method before progression. Therefore, identifying potential diagnostic and prognostic biomarkers for cancer patients to guide clinical decision is crucial and necessary.

Long noncoding RNAs (LncRNA) are a class of non-protein coding RNA molecules with more than 200 nucleotides in length, which were considered as genomic “junk” and “noise” initially [3]. The advancement of genome sequencing technology enabled an unprecedented exploration of genomic landscape for LncRNAs [4]. Recent studies indicate that aberrant lncRNA expression is involved in carcinogenesis, metastasis and progress, and its intimate association with tumor has rencently caught increasing attention [5, 6].

Colon cancer-associated transcript 2(CCAT2), a 1752-bp lncRNA transcribed from m8q24 genomic region, was first discovered by Ling et al. in 2013 [7]. They found that genomic locus of CCAT2 is highly conserved and harbors the SNP rs6983267.It was highly expressed in microsatellite-stable colorectal cancer and promotes tumor growth, metastasis, and chromosomal instability. Previous researchers showed that CCAT2 expression was highly expressed in various malignancies, such as breast cancer [8], gastric cancer [9], cervical cancer [10], esophageal cancer [11], ovarian cancer [12], and lung cancer [13]. Redis et al reported that CCAT2 migh be served as a poor prognosis marker in breast cancer patients. In addition, they found that CCAT2 upregulates cell migration and downregulates chemosensitivity to 5’FU in an rs6983267-independent manner [14]. Qiu et al found that silencing CCAT2 led to inhibition of proliferation and invasion in NSCLC,and CCAT2 could predict lymph node metastasis(LNM) [15]. Wang et al observed that high lncRNA CCAT2 expression was correlated with lymph node metastasis and distance metastasis (DM)in gastric cancer [11].Wu et al found that knockdown of CCAT2 can inhibit cervical cancer cell proliferation and promote cervical cancer cells apoptosis [16]. These studies suggest that CCAT2 gene may act as a novel oncogene in these tumors,regulating celluar growth,migration and apoptosis. Moreover, abnormal expression of CCAT2 was suggested to be associated with lymph node metastasis,distant metastasis and overall survival [17] . Therefore, we performed this meta-analysis to investigate the lymph node metastasis, distant metastasis and clinical prognostic role of overexpression CCAT2 in human cancers.

## RESULT

### Characteristics of eligible studies

**Figure 1:**
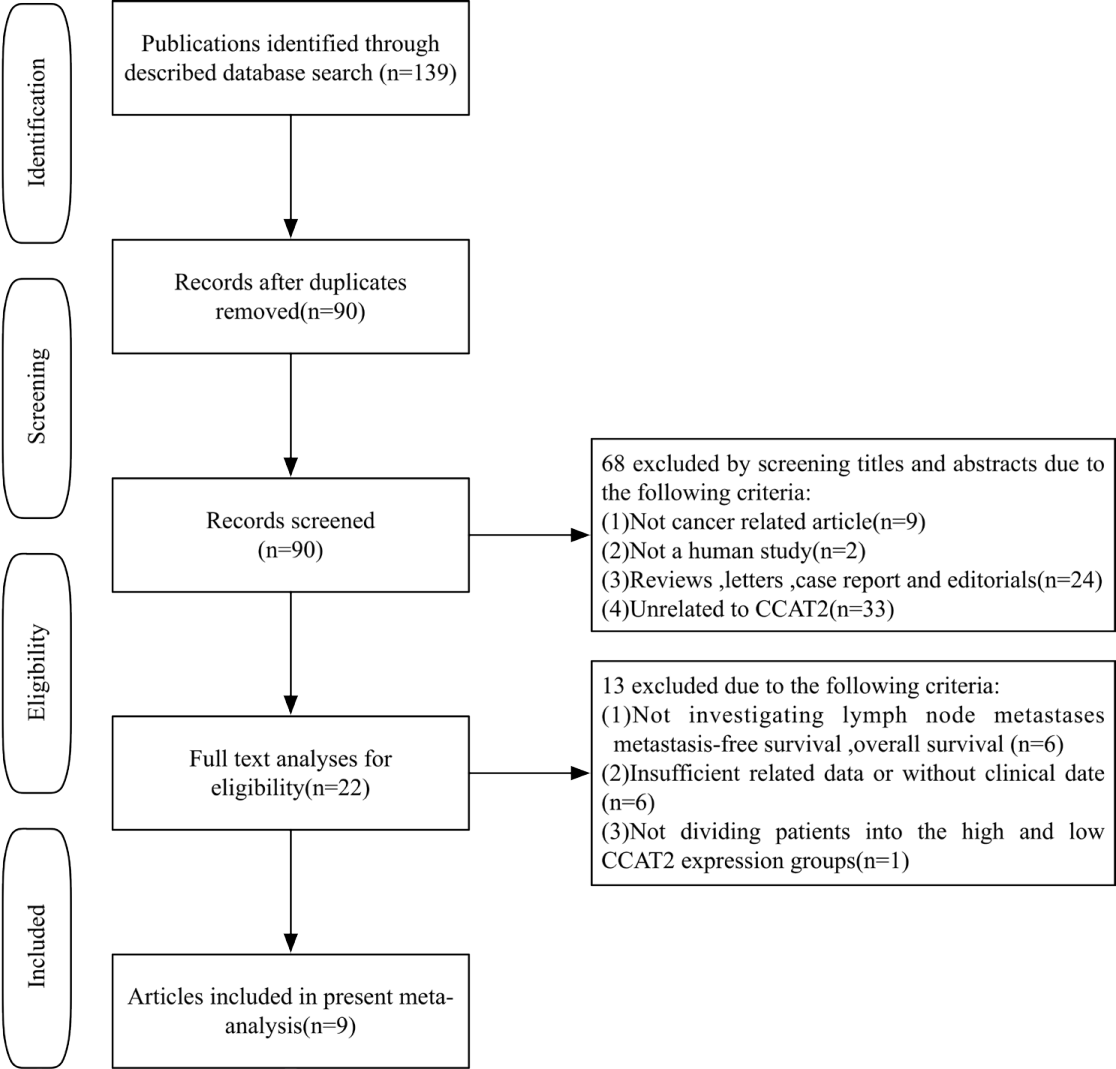
The flow diagram of this meta-analysis

As shown in the flow diagram (Figure 1), a total of 139 published records were conducted in our initial search and 49 duplicate articles were exclued. After screening of the title and abstract carefully, 68 irrelevant articles were excluded and 22 potential eligible studies were selected. After further review of the full articles, 13 papers were eliminated due to lack of information regarding LNM,DM or survival outcomes. Finally, according to the criteria for selection, a total of 9 studies were eligible for further research [7, 8, 10-14, 18, 19].

A total of 1084 patients were included, with the sample size ranging from 67 to 229. The period of these studies were published between 2013 and 2016.Seven included studies were from China, and the other two were from Netherlands. Among the nine studies, three focused on breast cancer(BC), one on gastric cancer(GC), one on cervical squamous cell (CSC), one on esophageal squamous cell carcinoma(ESCC) , one on ovarian cancer(OC) ,one on small cell lung cancer (SCLC) ,and one on prostate cancer(Pra).The expression of CCAT2 was detected by qRT-PCR and normalized to GAPDH or β-actin.In all of the studies, the patients were divided into two groups: high and low expression of CCAT2.HR and its 95%CI were extracted directly from original data reported in the papers. All diagnoses of LNM and DM were based on pathology. The main characteristics of the eligible studies were summarized in Table-1. The Newcastle-Ottawa Scale (NOS) confirmed that all the studies were of high quality (Table-2).

**Table 1:** Characteristics of studies in this meta-analysis.

Study	Year	Country	Cancer type	Total number	Detection method	Cut-off	CCAT2 expression						Survival analysis	Multivariate analysis	HR statistic	Hazard ratios (95%CI)	Follow-up months
							High	High with DM	High with LNM	Low	High with DM	Low with LNM					
Ling	2013	Nertherlands	BC	129	RT-qPCR	X-tilealgorithm	68	NA	NA	61	NA	NA	MFS	NO	Rep	2.01(1.20-3.35)	120
Redis	2013	Netherlands	BC	134	RT-qPCR	Median	50	NA	NA	84	NA	NA	MFS	Yes	Rep	2.25(1.07-4.74)	60
Wang	2015	China	GC	85	RT-qPCR	ROC curve	44	11	28	41	3	8	OS	Yes	Rep	2.405(1.194-5.417)	60
Chen	2015	China	CSC	123	RT-qPCR	Median	62	NA	34	61	NA	11	OS	Yes	Rep	2.813(1.504-6.172)	60
Zhang	2015	China	ESCC	229	RT-qPCR	Median	115	NA	65	114	NA	48	OS	Yes	Rep	1.432 (1.005–2.041)	66
Cai	2015	China	BC	67	RT-qPCR	X-tilealgorithm	25	NA	NA	42	NA	NA	OS	Yes	SC	5.92(1.92-18.27)	60
Huang	2016	China	OC	109	RT-qPCR	Median	55	36	NA	54	6	NA	OS	No	Rep	2.938(1.526-5.873)	60
Chen	2016	China	SCLC	112	RT-qPCR	Median	56	14	45	56	0	22	OS	Yes	Rep	2.034(1.216-3.402)	60
Zheng	2016	China	PC	96	RT-qPCR	Median	59	32	10	37	8	5	OS	No	Rep	2.292(1.370-3.528)	60

BC breast cancer, GC gastric cancer, CSC cervical squamous cell cancer, ESCC esophageal squamous cell carcinoma, OC ovarian cancer, SCLC small cell lung cancer, PC prostate cancer, DM distant metastasis, LNM: lymph node metastasis,RT-qPCR quantitative real-time PCR,NA not available, Rep reported,SC survival curve,OS overall survival, MFS metastasis-free survival, ROC curve: receiver operative curve.

### Meta-analysis results

#### Association between CCAT2 and LNM

**Figure 2:**
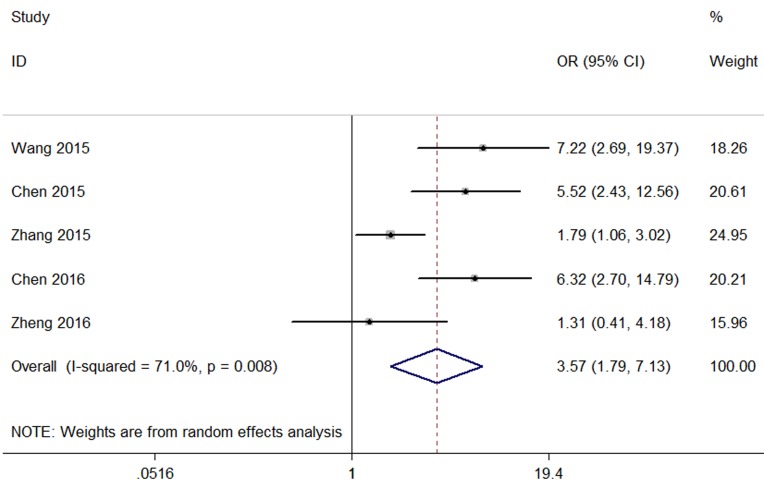
Forest plot for the association between CCAT2 expression levels with LNM

Five studies reporting a total of 645 patients with LNM were included based on different CCAT2 expression levels. The random-effects model was adopted as the significant heterogeneity (I^2^=71%, p=0.008). Analysis showed that the OR was3.57 with 95 % CI: 1.79-7.13 (p<0.001) (Figure 2)

Because of the existence of heterogeneity (I^2^=71%, p=0.008),we performed a sensitivity analysis to clarify the possible source of heterogeneity.After excluding the Zhang study[18],the heterogeneity obviously declined(I^2^ =49.9%, P =0.112) and the results did not change (OR=4.59, 95 % CI:2.36-8.93, P<0.001) (Sup-1,2).The results also revealed a significant difference in the LNM incidence between the two groups.Therefore, the results demonstrated that high CCAT2 expression might significantly predict a higher tendency to develop LNM in patients with cancer.

#### Association between CCAT2 and DM

**Figure 3:**
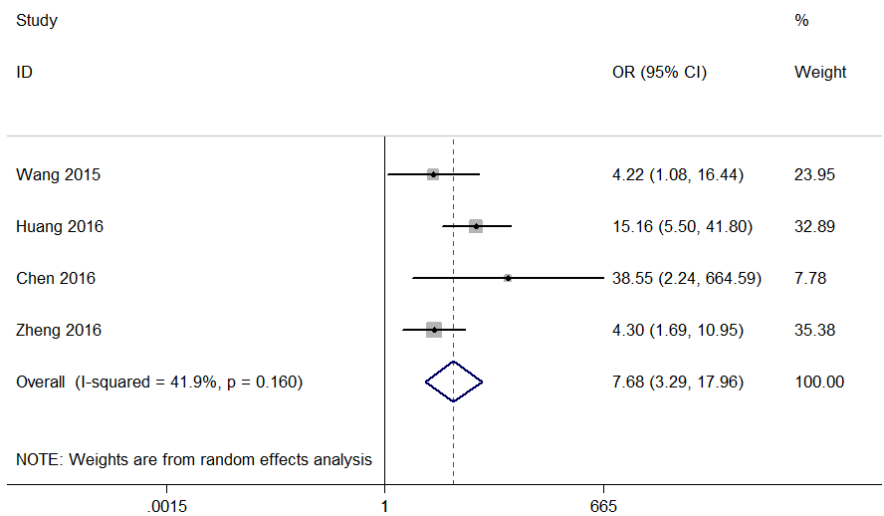
Forest plot of the association between CCAT2 expression levels and DM

Four studies reported the DM of 402 patients based on different CCAT2 expression levels. Analysis showed a pooled OR=7.68 (95 %CI:3.29–17.96, p<0.001) in a random-effects model (I^2^=41.9%,P=0.16) (Figure 3). The result indicated that overexpression of CCAT2 was significantly related to DM.

#### Association between CCAT2 and metastasis-free survival

**Figure 4:**
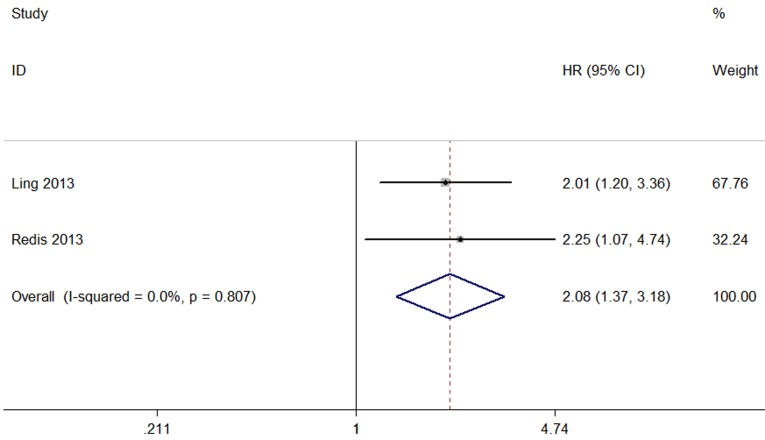
Forest plot showing the association between MFS and CCAT2 expression in cancer

A total of 263 patients in two studies were included to detect the relationship between CCAT2 expression levels and metastasis-free survival (MFS) in this meta-analysis. We observed a significant association between MFS and CCAT2 expression (HR= 2.08, 95% CI: 1.37–3.18 P=0.001) (Figure 4). In addition, we did not observe any significant heterogeneity among the studies (I^2^ =0.0%, P =0.807). This result demonstrated that high CCAT2 expression might be significantly associated with shorter MFS.

#### Association between CCAT2 and OS

**Figure 5:**
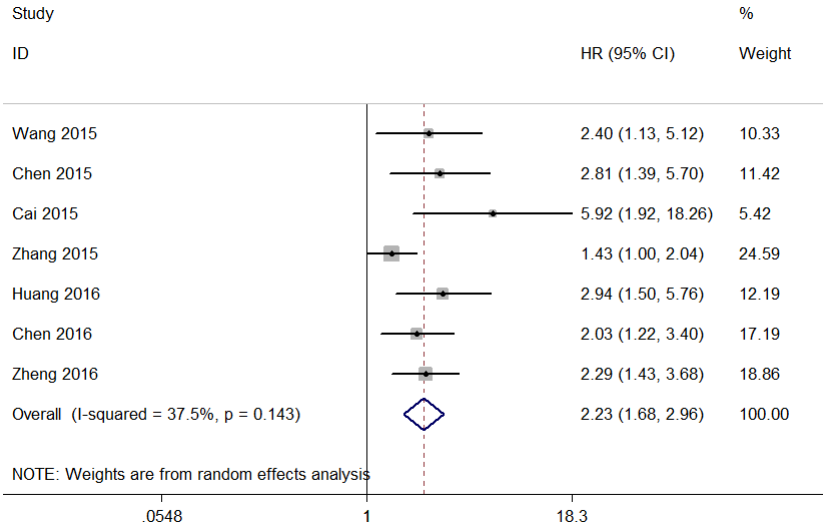
Forest plot of the pooled HRs of elevated CCAT2 expression for OS in different cancer patients

Data of pooled HRs and 95 %CI of OS were collected from the seven eligible studies. Analysis showed a pooled HR of 2.23 with(95 %CI 1.68–2.96,P<0.00001) by a random-effects model (I^2^=37.5%, p=0.143) (Figure 5).

**Figure 6:**
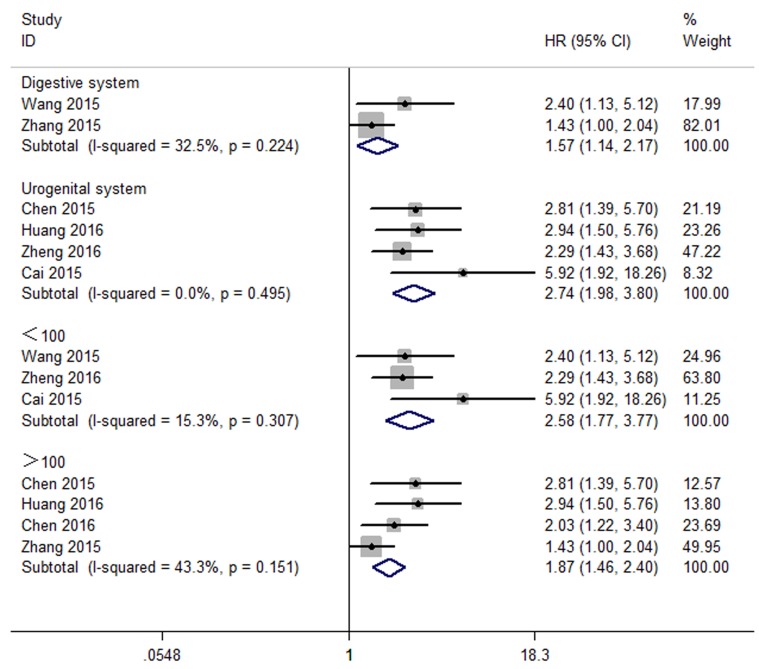
Forest plot showing the subgroup analyses of the pooled HRs of OS with elevated CCAT2 expression in different cancer types

Subgroup analysis was performed to explore the sources of heterogeneity. In a subgroup analysis of cancers type,significant association was found between high expression levels of CCAT2 and OS in the digestive system cancers(HR: 1.57, 95%CI: 1.14-2.17, P=0.006) and urogenital system cancer patients (HR=2.74,95% CI: 1.98–3.80, p<0.0001).The association between CCAT2 and OS was significant in studies with sample size both fewer than 100 (HR:2.58, 95%CI: 1.77-3.77, P<0.001) and more than 100 (HR:1.87,95%CI:1.46-2.40,P<0.001).And there was no significant heterogeneity in this subgroup annalysis. (Figure 6).

According to these results, we found that overexpression of CCAT2 could predict poor OS in various cancers (Table 3).

**Table-2:** Quality assessment of eligible studies (Newcastle-Ottawa Scale).

Study	Selection			Comparability			Outcome		Total
	Adequacy of case definition	Number of case	Representativeness of the cases	Ascertainment of exposure	Ascertainment of detection method	Ascertainment of cut-off	Assessment of outcome	Adequate follow up	
Ling 2013	1	1	1	1	1	1	1	1	8
Redis 2013	1	1	1	1	1	1	1	1	8
Wang 2015	1	1	1	1	1	1	1	1	8
Chen 2015	1	1	1	1	1	1	1	1	8
Zhang 2015	0	1	1	1	1	1	1	1	7
Cai 2015	1	1	1	1	1	1	1	0	7
Huang 2016	0	1	1	1	1	1	1	1	7
Chen 2016	1	1	1	1	1	1	1	0	7
Zheng 2016	1	1	1	1	1	1	1	1	8

**Table 3:** Results of this meta-analysis.

Outcome	No.of studies	No.of patients	HR/OR(95%CI)	*P*	Heterogeneity	
					*I*^2^(%)	*p*-value
OS	7	821	2.06 (1.67-2.54)	<0.00001	37.5	0.143
Cancer type						
Digestive system	2	314	1.57(1.14-2.17)	0.006	32.5	0.224
Urogenital system	4	395	2.74(1.98-3.80)	<0.0001	0.00	0.495
Sample size						
<100	3	248	2.58(1.77-3.77)	<0.001	15.3	0.307
<100	4	573	1.87(1.46-2.40)	<0.001	31.8	0.221
MFS	2	263	2.08(1.37-3.18)	0.001	0.00	0.807
LNM	4	416	4.59(2.36-8.93)	<0.001	49.9	0.112
DM	4	402	7.68(3.29-17.96)	<0.001	41.9	0.160

OS: overall survival, MFS: metastasis-free survival, LNM: lymph node metastasis, DM: distant metastasis ,HR: hazard ratios, OR: odds ratios, No: number, CI: confidence interval.

### Publication bias and sensitivity analysis

**Figure 7:**
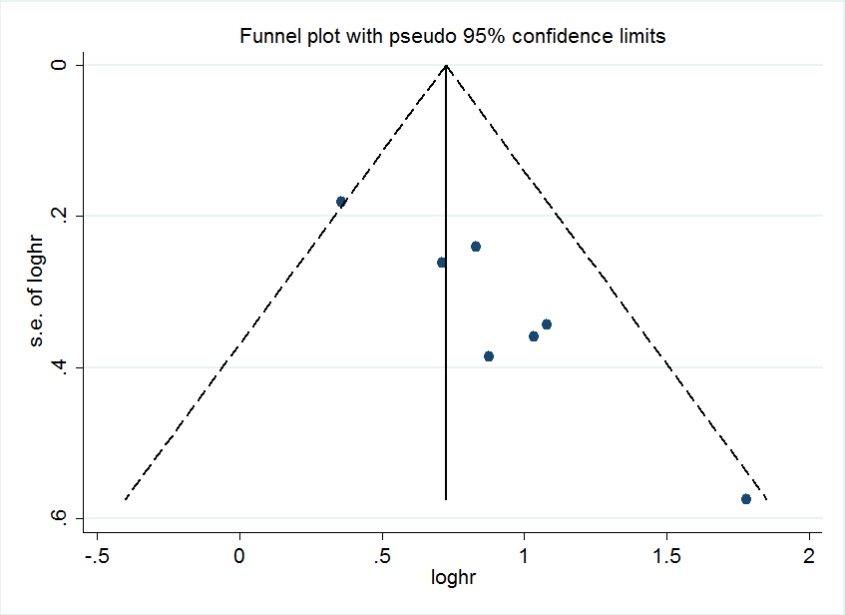
Funnel plot of the publication bias for the analysis of the independent role of CCAT2 in OS in the different cancer types

**Figure 8:**
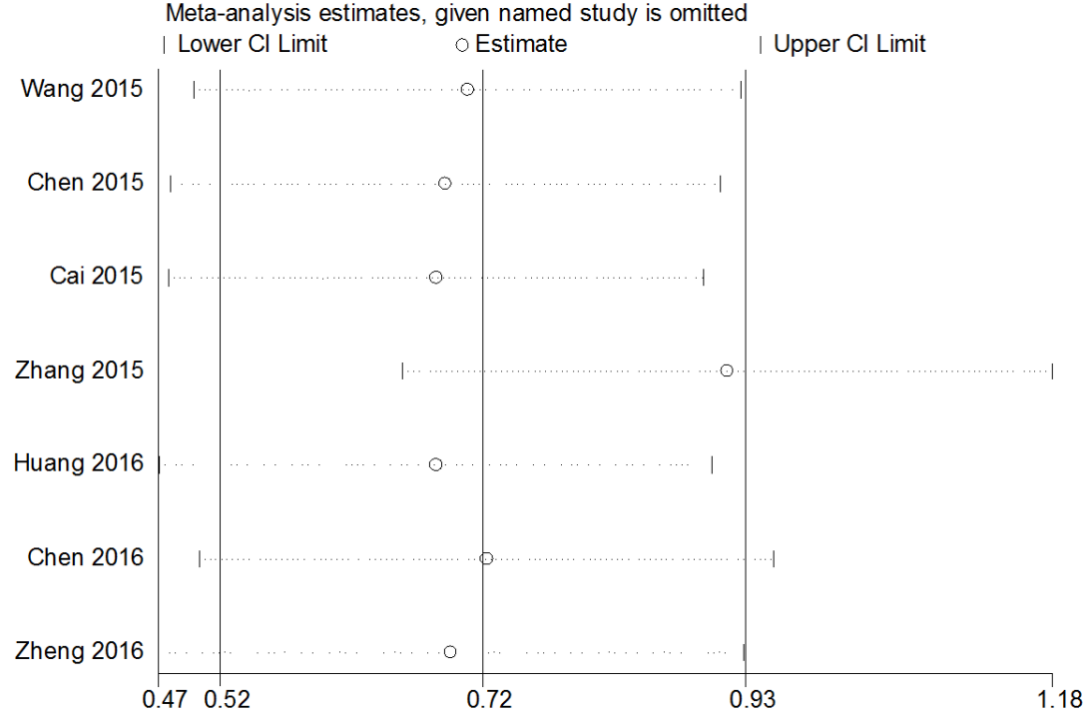
Sensitivity ananlysis of effect of individual studies on the pooled CCAT2 and OS of patients

The publication bias of the present meta-analysis was evaluated by funnel plot and Egger’s test. The shape of the funnel plot was almost symmetrical; it did not reveal any evidence of obvious asymmetry (Figure 7). As for LNM,DM and MFS groups, the publication bias was not analyzed because of the small number of studies. To test the stability of meta-analysis of CCAT2 and OS, we performed sensitivity analysis by sequentially removing each eligible study and result pattern was not significantly impacted (Figure 8).

## DISCUSSION

LncRNA CCAT2 which has been detected to be overexpressed in many malignancies may act as a novel oncogene [7, 8]. The genomic locus of CCAT2 is highly conserved and harbors the SNP rs6983267, which was shown to be associated with risk of several cancers, such as colorectal, prostate, and thyroid [20-23] .It was previously demonstrated that CCAT2 promotes cell growth and chromosomal instability in several malignant tumors through MYC-regulated miRNAs miR-17-5p and miR-20a and other pro-metastatic targets including CDC25A.[24] Moreover, upregulation of CCAT2 can promote the proliferation and metastasis of cancer cells by enhancing the WNT pathway [25]. Furthermore, CCAT2 has been shown to physically interact with TCF7L2, by which the expressions of MYC, miR-17-5p, and miR-20a are up-regulated, resulting in activation of the WNT signaling pathway [7, 8, 26].CCAT2 also appears to be involved in migration and chemoresistance in a SNP-independent fashion in breast cancer [14]. However, further investigation is needed to explain the molecular mechanisms of CCAT2 in tumor development.

This meta-analysis amied to explore the association between the expression level of CCAT2 and tumor metastasis & prognosis. To our knowledge, this is the first meta-analysis providing comprehensive insights into the clinical value of lncRNA CCAT2 in cancers. Our analysis indicated that overexpression of CCAT2 was significant correlated with LNM (OR=3.57, 95 %(CI): 1.79-7.13, p<0.001). Moreover, we observed that high CCAT2 expression was significantly correlated with DM(OR=7.68, 95 % CI: 3. 29-17.96, p<0.001) .

There was substantial heterogeneity between CCAT2 expression level and LNM. Heterogeneity is a potential problem in interpreting the results of any meta-analysis. To resolve this problem, we performed a sensitivity analysis,and the existing heterogeneity obviously declined (I^2^ =49.9%, P =0.112) after excluding the Zhang study [18], but the results did not change (OR=4.59, 95 % CI:2.36-8.93, P<0.001).This means Zhang study might have a significant influence on overall heterogeneity. Through our further analysis, we thought the potential sources of heterogeneity for this result may include:(1) The sample size and sample selection of this study may influence the heterogeneity. (2) In included studies, RT-qPCR reaction conditions and reaction systems were different, and this difference may affect the results. (3) The characteristics of different tumor clinical pathological parameters might lead to potential heterogeneity. In this paper, the pathological type of esophageal squamous cell carcinoma may result in heterogeneity. (4) The inconsistent cut-off value for CCAT2 in this paper might be ralated to the heterogeneity.

Furthermore,by combining HRs from the Cox multivariate analyses, we observed that CCAT2 was an independent prognostic factor of OS for cancer patients (pooled HR 2.23 [95 % CI 1.68–2.96,P<0.00001]). In the subgroup analysis, a significant association between CCAT2 and OS in digestive system (HR: 1.57, 95%CI: 1.14-2.17, P=0.006) and urogenital system cancer patients (HR=2.74,95% CI: 1.98-3.80, p<0.0001) was observed. The association between CCAT2 and OS was significant in studies with sample size both fewer than 100 (HR:2.58, 95%CI: 1.77-3.77, P<0.001) and more than 100 (HR:2.612,95%CI:1.949-3.499, P<0.001).

Additionally, a significant association between MFS and CCAT2 expression (HR:1.87,95%CI:1.46-2.40, P<0.0001) was also observed.

Through the above analyses, we found that high expression of CCAT2 predicted more prone to LNM, DM and poor survival outcome. However, several limitations in the present meta-analysis should be emphasized. First, all of the included articles were retrospective and most of them had a small sample size. Only 9 studies with 7 types of tumor were included in this meta-analysis; Second, different types of cancers increased the heterogeneity and inherent molecular differences might influence accuracy of the results. Third, cut-off values were inconsistent among studies which may reduced the power for detecting a real association. Fourth, many included studies reported significant results because works with non-significant results would have little chance to be published.

In conclusion,despite the limitations described above,the meta-analysis offers evidence that the expression levels of CCAT2 is significantly associated with LNM,DM and survial outcome in cancer patients. Moreover, CCAT2 might serve as a potentially molecular marker for LNM, DM, OS and MFS in human cancers. However, more clinical studies and better design studies are needed to elucidate and confirm the findings of this analysis.

## MATERIALS AND METHODS

### Literature search

Electronic search was perfromed in PubMed, Cochrane Library, and Web of Science, using “lncRNA CCAT2” or “Colon cancer-associated transcripts2” as the keywords. Citation lists of retrieved articles were searched manually to ensure eligibable studies. The covered literatures were restricted to publications in English. The last update of searching time was NOV 17, 2016.

### Inclusion and exclusion criteria

Inclusion criteria are as the following: (1) studies exploring the clinical roles of CCAT2 in various cancers. (2) patients were grouped according to the expression levels of CCAT2.(3) related clinicopathologic parameters were described.(4) reporting of clinical data, including lymph node metastasis (LNM) ,distant metatasis(DM), overall survival (OS), and metastasis-free survival(MFS) . (5) studies containing sufficient data for the computation of odds ratios (OR) and corresponding 95 % confidence intervals (CI) or a P. value.Studies were excluded based on the following criteria: (1) studies investigating the molecular structure and functions of CCAT2 (2) duplicate publications, nonhuman research, letters, editorials, expert opinions, case reports and reviews. (3) lack of usable data.

### Date extraction

Data extraction was performed independently by three authors (WDL, CZC and HAB). Disagreements were discussed with two investigators (LYC and HWR) by discussions. According to the inclusion and exclusion criteria, the following information were recorded: (1) first author;(2) publication year; (3)country of origin;(4) tumor type;(5) sample size,;(6) number of high CCAT2 expression group and low CCAT2 expression group;(7) CCAT2 assessment methods;(8) follow-up period and cut-off values ;(9) ORs of CCAT2 for LNM and DM: number of patients with LNM and DM in each group; (10)HRs, and corresponding 95% CIs for OS,PFS or MFS . Disagreements were resolved by discussions on all of the items.

The study quality was assessed in accordance with the NOS. Nine items were extracted, and each item scored 1. The total scores ranged from 0 to 9. If the scores were≥7, then the study was considered high quality (Table.2).

### Statistical analysis

Pooled hazard ratios (HRs) and its 95% CI were collected from the included studies if they were reported in the articles,while they were calculated with available data, such as the exact patient numbers and Kaplan–Meier(K–M) curve by the method from Parmar et al [27].The log HR and standard error (SE) were used for aggregation of the survival results[28, 29]. In order to evaluate the heterogeneity of the eligible studies, pooled HRs were executed using I^2^ statistics in this meta-analysis [30]. If there was a significant statistical in the heterogeneity among the studies (P<0.1), the random-effects model was odopted to analyze the results, and if not, the fixed-effects model was used (P>0.1).Forest plots were applied for displaying the meta-analysis results. The potential publication bias was assessed using the Egger’s test. All the statistical analyses were carried out by using the Stata 12.0. P values<0.05 were considered statistically significant.

## SUPPLEMENTARY MATERIALS FIGURES


