# Supplementary material for: Long noncoding RNA CCAT2 as a novel biomaker of metastasis and prognosis in human cancer: a meta-analysis
Source: Oncotarget. 2017 May 24;8(43):75664–74. doi: 10.18632/oncotarget.18161 (PMC5650455; doi:10.18632/oncotarget.18161)
Supplement: Supplementary file 1 [file oncotarget-08-75664-s001.pdf]

Long noncoding RNA CCAT2 as a novel biomaker of metastasis and prognosis in human cancer: a meta-analysis

SUPPLEMENTARY MATERIALS

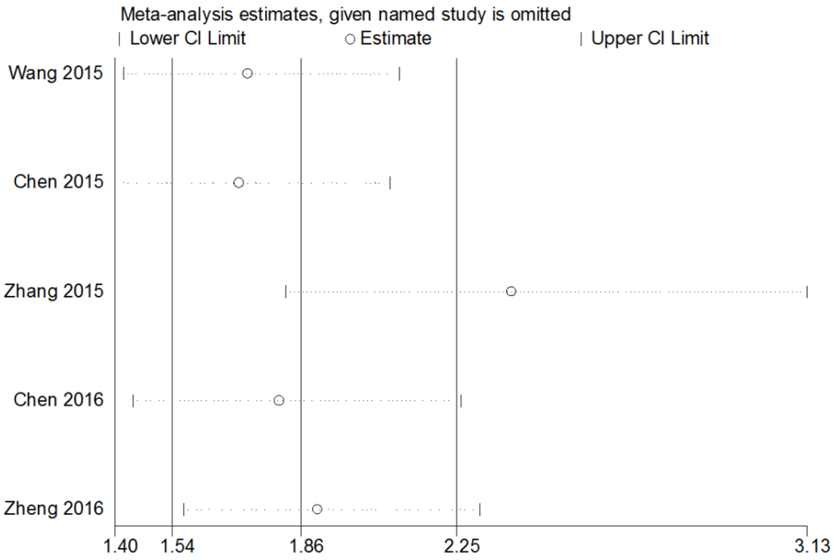

Supplementary Figure 1: Result of sensitivity analysis in LNM group.

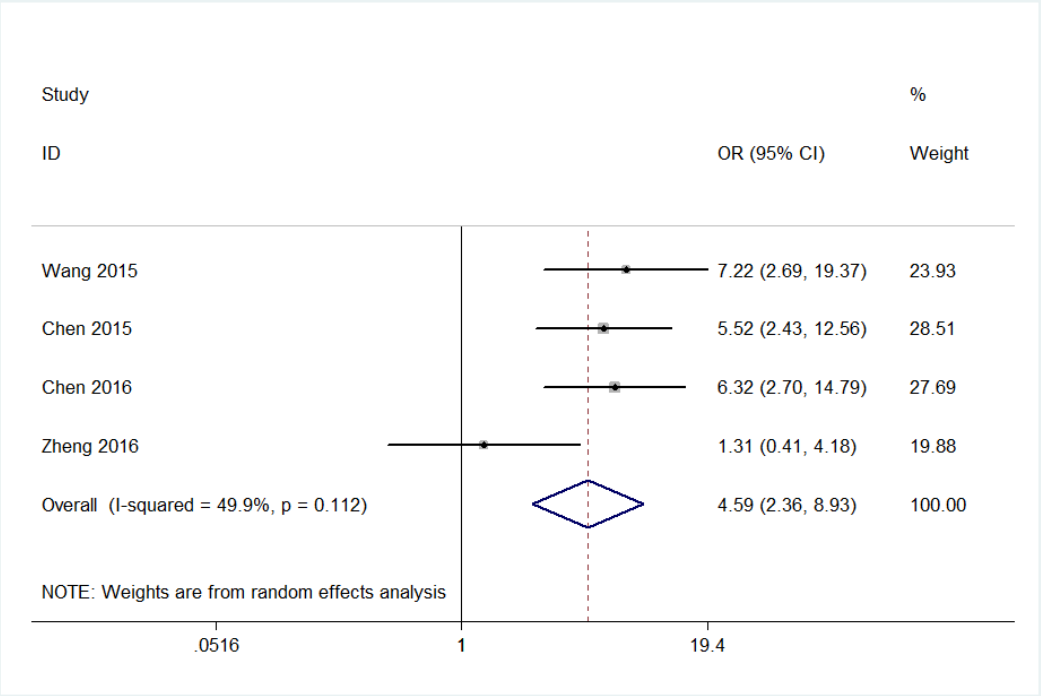

Supplementary Figure 2: Forest plot of the association between CCAT2 expression levels and LNM after after excluding one study.
